# Bonsai Trees in Your Head: How the Pavlovian System Sculpts Goal-Directed Choices by Pruning Decision Trees

**DOI:** 10.1371/journal.pcbi.1002410

**Published:** 2012-03-08

**Authors:** Quentin J. M. Huys, Neir Eshel, Elizabeth O'Nions, Luke Sheridan, Peter Dayan, Jonathan P. Roiser

**Affiliations:** 1 Gatsby Computational Neuroscience Unit, University College London, London, United Kingdom; 2 Wellcome Trust Centre for Neuroimaging, Institute of Neurology, University College London, London, United Kingdom; 3 Guy's and St. Thomas' NHS Foundation Trust, London, United Kingdom; 4 UCL Institute of Cognitive Neuroscience, London, United Kingdom; New York University, United States of America

## Abstract

When planning a series of actions, it is usually infeasible to consider all potential future sequences; instead, one must prune the decision tree. Provably optimal pruning is, however, still computationally ruinous and the specific approximations humans employ remain unknown. We designed a new sequential reinforcement-based task and showed that human subjects adopted a simple pruning strategy: during mental evaluation of a sequence of choices, they curtailed any further evaluation of a sequence as soon as they encountered a large loss. This pruning strategy was Pavlovian: it was reflexively evoked by large losses and persisted even when overwhelmingly counterproductive. It was also evident above and beyond loss aversion. We found that the tendency towards Pavlovian pruning was selectively predicted by the degree to which subjects exhibited sub-clinical mood disturbance, in accordance with theories that ascribe Pavlovian behavioural inhibition, via serotonin, a role in mood disorders. We conclude that Pavlovian behavioural inhibition shapes highly flexible, goal-directed choices in a manner that may be important for theories of decision-making in mood disorders.

## Author Summary

Planning is tricky because choices we make now affect future choices, and future choices and outcomes should guide current choices. Because there are exponentially many combinations of future choices and actions, brute-force approaches that consider all possible combinations work only for trivially small problems. Here, we describe how humans use a simple Pavlovian strategy to cut an expanding decision tree down to a computationally manageable size. We find that humans use this strategy even when it is disadvantageous, and that the tendency to use it is related to mild depressive symptoms. The findings, we suggest, can be interpreted within a theoretical framework which relates Pavlovian behavioural inhibition to serotonin and mood disorders.

## Introduction

Most planning problems faced by humans cannot be solved by evaluating all potential sequences of choices explicitly, because the number of possible sequences from which to choose grows exponentially with the sequence length. Consider chess: for each of the thirty-odd moves available to you, your opponent chooses among an equal number. Looking 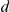 moves ahead demands consideration of 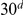 sequences. Ostensibly trivial everyday tasks, ranging from planning a route to preparing a meal, present the same fundamental computational dilemma. Their computational cost defeats brute force approaches.

These problems have to be solved by pruning the underlying decision tree, i.e.by excising poor decision sub-trees from consideration and spending limited cognitive resources evaluating which of the good options will prove the best, not which of the bad ones are the worst. There exist algorithmic solutions that ignore branches of a decision tree that are guaranteed to be worse than those already evaluated [1]–[3]. However, these approaches are still computationally costly and rely on information rarely available. Everyday problems such as navigation or cooking may therefore force precision to be traded for speed; and the algorithmic guarantees to be replaced with powerful––but approximate and potentially suboptimal––heuristics.

**Figure 1:**
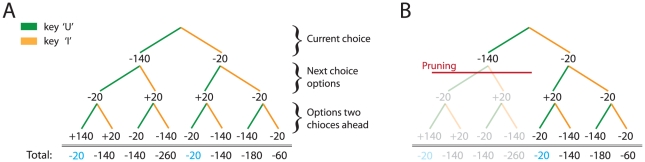
Decision tree. **A**: A typical decision tree. A sequence of choices between ‘U’ (left, green) and ‘I’ (right, orange) is made to maximize the total amount earned over the entire sequence of choices. Two sequences yield the maximal total outcome of −20 (three times U; or I then twice U). Finding the optimal choice in a goal-directed manner requires evaluating all 8 sequences of three moves each. **B**: Pruning a decision tree at the large negative outcome. In this simple case, pruning would still favour one of the two optimal sequences (yielding −20), yet cut the computational cost by nearly half.

Consider the decision tree in Figure 1A, involving a sequence of three binary choices. Optimal choice involves evaluating 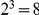 sequences. The simple heuristic of curtailing evaluation of all sequences every time a large loss (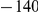) is encountered excises the left-hand sub-tree, nearly halving the computational load (Figure 1B). We term this heuristic pruning a “Pavlovian” response because it is invoked, as an immediate consequence of encountering the large loss, when searching the tree in one's mind. It is a reflexive response evoked by a valence, here negative, in a manner akin to that in which stimuli predicting aversive events can suppress unrelated ongoing motor activity [4], [5].

A further characteristic feature of responding under Pavlovian control is that such responding persists despite being suboptimal [6]: pigeons, for instance, continue pecking a light that predicts food, even when the food is omitted on every trial on which they peck the light [7], [8]. While rewards tend to evoke approach, punishments appear particularly efficient at evoking behavioral inhibition [9], [10], possibly via a serotonergic mechanism [11]–[15]. Here, we will ascertain whether pruning decision trees when encountering losses may be one instance of Pavlovian behavioural inhibition. We will do so by leveraging the insensitivity of Pavlovian responses to their ultimate consequences.

**Figure 2:**
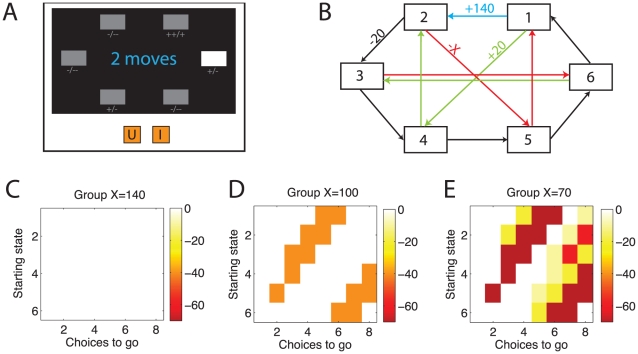
Task description. **A**: Task as seen by subjects. Subjects used two buttons on the keyboard (‘U’ and ‘I’) to navigate between six environmental states, depicted as boxes on a computer screen. From each state, subjects could move to exactly two other states. Each of these was associated with a particular reinforcement. The current state was highlighted in white, and the required sequence length displayed centrally. Reinforcements available from each state were displayed symbolically below the state, e.g. 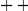 for the large reward. **B**: Deterministic task transition matrix. Each button resulted in one of two deterministic transitions from each state. For example, if the participant began in state 6, pressing ‘U’ would lead to state 3, whereas pressing ‘I’ would lead to state 1. The transitions in red yielded large punishments. These (and only these) differed between three groups of subjects (−140, −100 or −70). Note that the decision trees in Figure 1A,B correspond to a depth 3 search starting from state 3. **C–E**: Effect of pruning on values of optimal choices. Each square in each panel analyses choices from one state when a certain number of choices remains to be taken. The color shows the difference in earnings between two choice sequences: the best choice sequence with pruning and the best choice sequence without pruning. In terms of net earnings, pruning is never advantageous (pruned values are never better than the optimal lookahead values); but pruning does not always result in losses (white areas). It is most disadvantageous in the −70 group, and it is never disadvantageous in the −140 group because there is always an equally good alternative choice sequence which avoids transitions through large losses.

We developed a sequential, goal-directed decision-making task in which subjects were asked to plan ahead (c.f. [16]). On each trial, subjects started from a random state and generated a sequence of 2–8 choices to maximize their net income (Figure 2A,B). In the first of three experimental groups the heuristic of pruning sub-trees when encountering large punishments incurred no extra cost (Figure 2C). Subjects here pruned extensively: they tended to ignore subtrees lying beyond large losses. This alleviated the computational load they faced, but did not incur any costs in terms of outcomes because there was always an equally good sequence which avoided large losses (see Figure 2C). In contrast, in the second and third experimental groups subjects incurred increasingly large costs for this pruning strategy (Figure 2D,E); yet, they continued to deploy it. That is, the tendency to excise subtrees lying below punishments persisted even when counterproductive in terms of outcomes. This persistence suggests that pruning was reflexively evoked in response to punishments and relatively insensitive to the ultimate outcomes.

Computational models which accounted for close to 90% of choices verified that the nature of pruning corresponded to the Pavlovian reflexive account in detail. These results reveal a novel type of interaction between computationally separate decision making systems, with the Pavlovian behavioural inhibition system working as a crutch for the powerful, yet computationally challenged, goal-directed system. Furthermore, the extent to which subjects pruned correlated with sub-clinical depressive symptoms. We interpret this in the light of a theoretical model [17] on the involvement of serotonin in both behavioural inhibition [14], [15] and depression.

## Results

**Figure 3:**
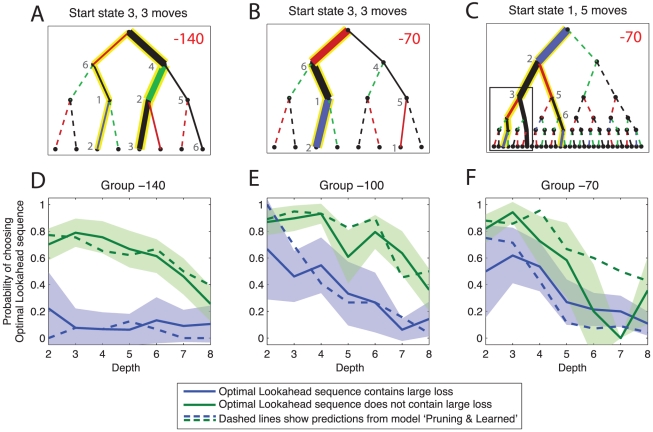
Choice sequences. Example decision trees of varying depth starting from states 1 or 3. The widths of the solid lines are proportional to the frequencies with which particular paths were chosen (aggregated across all subjects). Yellow backgrounds denote optimal paths (note that there can be multiple optimal paths). Colours red, black, green and blue denote transitions with reinforcements of 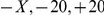 and 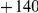 respectively. Dashed lines denote parts of the decision tree that were never visited. Visited states are shown in small gray numbers where space allows. **A**: Subjects avoid transitions through large losses. In the 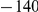 condition, this is not associated with an overall loss. **B**: In the 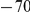 condition, where large rewards lurk behind the 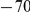 losses, subjects can overcome their reluctance to transition through large losses and can follow the optimal path through an early large loss. **C**: However, they do this only if the tree is small and thus does not require pruning. Subjects fail to follow the optimal path through the same subtree as in B (indicated by a black box) if it occurs deeper in the tree, i.e. in a situation where computational demands are high. **D,E,F** Fraction of times subjects in each group chose the optimal sequence, deduced by looking all the way to the end of the tree. Green shows subjects' choices when the optimal sequence did not contain a large loss; blue shows subjects' choices when the optimal sequence did contain a large loss. Coloured areas show 95% confidence intervals, and dashed lines predictions from the model ‘Pruning & Learned’ (see below).

Figure 3 shows representative decision paths. Figure 3A shows the decision tree subjects faced when starting from state 3 and asked to make a 3-step decision. In the −140 group, there are two equally good choice sequences in this situation: either through states 3-4-2-3 (with returns 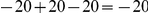 net) or through states 3-6-1-2 (with returns 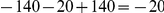 net). When given the choice, subjects reliably chose the path avoiding the large loss (even though this meant also avoiding the equally large gain). However, Figure 3B shows that subjects could overcome the reflexive avoidance of the large loss. In this situation, because the large loss is much smaller (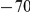), it is best to transition through it to reap the even larger reward (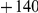) behind it. This same behaviour was less frequently observed in larger trees when large losses happened deeper in the tree. Figure 3C shows the tree of depth 5 starting from state 1. The leftmost three-move subtree, highlighted by the box, is identical to the tree starting from state 3 with depth 3. Although it is still optimal to transition through the large loss, subjects tended to avoided this transition and thereby missed potential gains. Note that in 3C, subjects also avoided an alternative optimal path where the large loss again did not occur immediately.

Figure 3D–F shows the number of times subjects chose the optimal sequence through the decision tree, separating out situations when this optimal choice involved a transition through a large loss and when it did not. Subjects were worse at choosing optimal sequences when the depth was greater. Subjects were also less willing to choose *optimal* sequences involving transitions through large losses (shown in blue) than those that did not (shown in green). This appeared to be the case more in the group −140 than the two other groups. However, this statistic is difficult to interpret because in this group there was always an optimal sequence which avoided the large loss. Nevertheless, we separated the blue traces into those cases where large losses appeared early or deep in the tree. For sequences of length 4 or 5, subjects were more likely to choose the optimal sequence if the loss appeared in the first rather than in the second half of the sequence (t-tests, 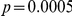 and 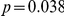 respectively). At depth of 6 or more there was no difference, but the number of these events was small, limiting the power.

Given these patterns in the data, we considered that subjects made goal-directed decisions [18] by evaluating decision paths sequentially. We directly tested the hypothesis whether they would avoid paths involving losses by terminating this sequential evaluation when encountering large losses. That is, in Figure 3C, do subjects neglect the large reward behind the large loss because they did not even consider looking past the large loss? Important alternative accounts (which the analyses so far do not fully address) are a simple inability to look so far ahead in this task (“discounting”), an overweighting of losses relative to rewards (“loss aversion”), and interference by other, non goal-directed, decision making strategies (“conditioned attraction & repulsion”). We assessed whether subjects' decision and inference strategies showed evidence of pruning by fitting a series of increasingly complex models assessing all these factors explicitly and jointly. This allowed a quantitative comparison of the extent to which the various hypotheses embodied by the models were able to account for the data.

### Decision making structure

**Figure 4:**
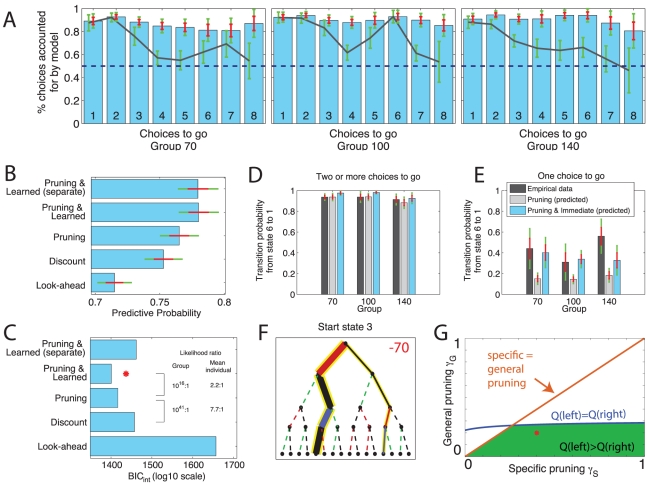
Model performance and comparison. **A**: Fraction of choices predicted by the model as a function of the number of choices remaining. For bars ‘3 choices to go’, for instance, it shows the fraction of times the model assigned higher 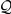 value to the subject's choice in *all* situations where three choices remained (i.e. bar 3 in these plots encompasses all three panels in Figure 3A–C). These are predictions only in the sense that the model predicts choice 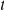 based on history up to 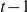. The gray line shows this statistic for the full look-ahead model, and the blue bars for the most parsimonious model (‘Pruning and Learned’). **B**: Mean predictive probabilities, i.e. likelihood afforded to choices on trial 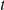 given learned values up to trial 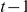. **C**: Model comparison based on integrated Bayesian Information Criterion (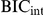) scores. The lower the 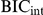 score, the more parsimonious the model fit. For guidance, some likelihood ratios are displayed explicitly, both at the group level (fixed effect) and at the individual level (random effect). Our main guide is the group-level (fixed effect). The red star indicates the most parsimonious model. **D,E**: Transition probability from state 6 to state 1 (which incurs a −20 loss) when a subsequent move to state 2 is possible (D; at least two moves remain) or not (E; when it is the only remaining move). Note that subjects' disadvantageous approach behavior in E (dark gray bar) is only well accommodated by a model that incorporates the extra Learned Pavlovian parameter. **F**: Decision tree of depth 4 from starting state 3. See Figure 3 for colour code. Subjects prefer (width of line) the optimal (yellow) path with an early transition through a large loss (red) to an equally optimal path with a late transition through a large loss. **G**: Phase plane analysis of specific and general pruning. Parameter values for which the left optimal yellow path in panel F is assigned a greater expected value than the right optimal path are below the blue line. Combinations that are also consistent with the notion of pruning 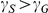 are shown in green. The red dot shows parameters inferred for present data (c.f. Figure 6). Throughout, errorbars indicate one standard error of the mean (red) and the 95% confidence intervals (green).

The first model ‘Look-ahead’ embodied full tree evaluation, without pruning. It assumed that, at each stage, subjects evaluated the decision tree all the way to the end. That is, for an episode of length 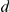, subjects would consider all 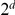 possible sequences, and choose among them with probabilities associated monotonically with their values. This model ascribed the higher action value to the subjects' actual choices a total of 77% of the time (fraction of choices predicted), which is significantly better than chance (fixed effect binomial 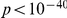). The gray lines in Figure 4A separate this by group and sequence length. They show that subjects in all three groups chose the action identified by the full look-ahead model more often than chance, even for some very deep searches. Figure 4B shows the predictive probability, i.e. the probability afforded to choices by the model. This is influenced by both the fraction of choices predicted correctly and the certainty with which they were predicted and took on the value 0.71, again different from chance (fixed effect binomial 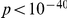). These results, particularly when considered with the fact that on half the trials subjects were forced to choose the entire sequence before making any move in the tree, indicate that they both understood the task structure and used it in a goal-directed manner by searching the decision tree.

In order to directly test hypotheses pertaining to pruning of decision trees, we fitted two additional models to the data. Model ‘Discount’ attempted to capture subjects' likely reluctance to look ahead fully and evaluate all sequences (up to 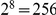). Rather, tree search was assumed to terminate with probability 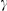 at each depth, substituting the value 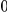 for the remaining subtree. In essence, this parameter models subjects' general tendency not to plan ahead. Figure 4B shows that this model predicted choices better. However, since an improved fit is expected from a more complex model, we performed Bayesian model comparison, integrating out all individual-level parameters, and penalizing more complex models at the group level (see Methods). Figure 4C shows that fitting this extra parameter resulted in a more parsimonious model. Note that this goal-directed model also vastly outperformed a habitual model of choice (SARSA; [19]) in which subjects are assumed to update action propensities in a model-free, iterative manner (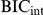 improvement of 314).

The third model, ‘Pruning’, is central to the hypothesis we seek to test here. This model separated subjects' global tendency to curtail the tree search (captured by the 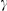 parameter of model ‘discount’) into two separate quantities captured by independent parameters: a general pruning parameter 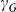, and a specific pruning parameter 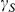. The latter applied to transitions immediately after large punishments (red ‘−X’ in Figure 2B), while the former applied to all other transitions. If subjects were indeed more likely to terminate their tree search after transitions resulting in large punishments, then a model that separates discounting into two separate pruning parameters should provide a better account of the data. Again, we applied Bayesian model comparison and found strong evidence for such a separation (Figure 4C).

The fourth model added an immediate Pavlovian influence on choice. The need for this can be seen by comparing the observed and predicted transition (action) probabilities at a key stage in the task. Figure 4D shows the probability that subjects moved from state 6 to state 1 when they had two or more choices left. Through this move, subjects would have the opportunity to reap the large reward of 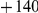 (see Figure 2B), by first suffering the small loss of −20. Subjects duly chose to move to state 1 on 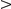
90% of these occasions in all three groups. This was well matched by the model ‘Pruning’. However, when subjects only had a single choice left in state 6, it would no longer be optimal to move to state 1, since there would be no opportunity to gain the large reward afterwards. Instead, the optimal choice would be to move to state 3, at a gain of 20. Despite this, on about 40% of such trials, subjects were attracted to state 1 (Figure 4E). This was not predicted by the pruning model: paired t-tests showed significant differences between empirical and predicted choice probabilities for each of the three groups: 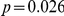, 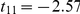; 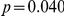, 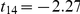; and 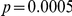, 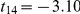, for groups −70, −100 and −140 respectively. Three subjects in group −70 and one subject in group −100 were never exposed to depth 1 sequences in state 6.

To accommodate this characteristic of the behavior, we added a further, ‘Learned Pavlovian’ component to the model, accounting for the conditioned attraction (or repulsion) to states that accrues with experience. This captured an immediate attraction towards future states that, on average (but ignoring the remaining sequence length on a particular trial), were experienced as rewarding; and repulsion from states that were, on average, associated with more punishment (see Methods for details). Figure 4B,C show that this model (Pruning and Learned) provided the most parsimonious account of the data despite two additional parameters, and Figures 4D–E show that the addition of the Learned parameters allowed the model to capture more faithfully the transition probabilities out of state 6. The blue bars in Figure 4A display the probability that this model chose the same action as subjects (correctly predicting 91% of choices). The model's predicted transition probabilities were highly correlated with the empirical choice probabilities in every single state (all 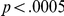). Further, we considered the possibility that the Learned Pavlovian values might play the additional role of substituting for the utilities of parts of a search tree that had been truncated by general or specific pruning. However, this did not improve parsimony.

We have so far neglected any possible differences between the groups with different large losses. Figures 3D–F might suggest more pruning in group −140 than in the other two groups (as the probability of choosing optimal full lookahead sequences containing a large loss is minimal in group −140). We therefore fitted separate models to the three groups. Figure 4B shows that the increase in the model flexibility due to separate prior parameters for each group (‘Pruning & Learned (separate)’) failed to improve the predictive probability, increased the 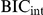 score (Figure 4C), and hence represents a loss of parsimony. Returning to Figure 3D–F, we plotted the predictions of model ‘Pruning & Learned’ for each of the three groups, and found that this model was able to capture the very extensive avoidance of optimal full lookahead sequences including large losses in group −140, and yet show a gradual decline in the other two groups.

The qualitative difference between group −140 and the two other groups in Figure 3D–F is also important because it speaks to the ‘goal-directed’ nature of pruning. Pruning is only counterproductive in groups −70 and −100. The apparent reduction in pruning suggested by the reduced avoidance of optimal sequences involving large losses in groups −70 and −100 (Figure 3E,F) could suggest that the extent of pruning depends on how adaptive it is, which would argue against a reflexive, Pavlovian mechanism. It is thus important that model ‘Pruning & Learned’ could capture these qualitative differences without recurrence to such a goal-directed, clever, pruning. It shows that these differences were instead due to the different reward structures (−70 is not as aversive as −140).

Finally, we return to the decision tree in Figure 3B. This would *prima facie* seem inconsistent with the notion of pruning, as subjects happily transition through a large loss at the very beginning of the decision sequence. Figure 4F shows a different facet of this. Starting from the state 3 again, subjects in group −70 choose the optimal path that goes through the large loss straight away even though there is an optimal alternative in which they do not have to transition through the large loss so early.

In fact, in the model, the relative impact of general and specific pruning factors interacts with the precise reinforcement sequence, and hence with the depth at which each reinforcement is obtained. More specifically, let us neglect the entire tree other than the two optimal (yellow) sequences the subjects actually took, and let 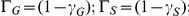. The value of the left sequence then equals 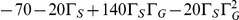. A similar, third-order polynomial in combinations of 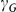 and 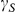 describes the value of the right path, and indeed their difference. The blue line in Figure 4G shows, for each value of 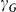, what value of 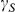 would result in the left and right sequences having the same value. The combinations of 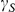 and 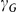 for which the chosen left path (with the early transition through the large loss) has a higher total value turn out to lie below this blue line. In addition, pruning will only be more pronounced after large losses if 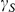 is larger than 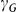. The overlap between these two requirements is shown in green, and the group means for 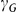 and 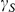 are shown by the red dot. Thus, because the effects of general and specific pruning interact with depth, the reflexive, but probabilistic, pruning in the model can lead to the pattern seen in Figure 4G, whereby subjects transition through large losses close to the root of the decision tree, but avoid doing so deeper in the tree. Put simply, fixed, reflexive Pavlovian pruning in these particular sequences of reinforcements has differential effects deep in the tree. In these cases, it matches the intuition that it is the exploding computational demands which mandate approximations. However, this is not a necessary consequence of the model formulation and would not hold for all sequences.

### Loss aversion

An alternative to the pruning account is the notion of loss aversion, whereby a loss of a given amount is more aversive than the gain of an equal amount is appetitive. Consider the following sequence of returns: 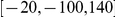 with an overall return of 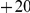. The pruning account above would assign it a low value because the large terminal gain is neglected. An alternative manner by which subjects may assign this sequence a low value is to increase how aversive a view they take of large losses. In this latter account, subjects would sum over the entire sequence, but overweigh large losses, resulting in an equally low value for the entire sequence.

**Figure 5:**
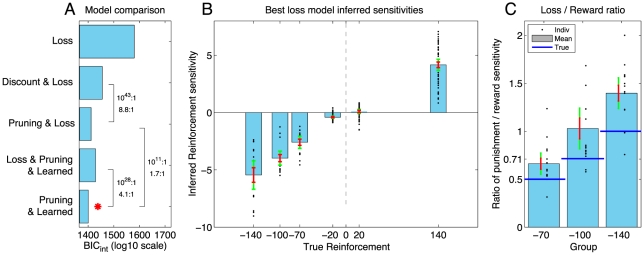
Pruning exists above and beyond any loss aversion. **A**: Loss aversion model comparison 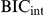 scores. Red star indicates most parsimonious model. The numbers by the bars show model likelihood ratios of interest at the group level, and below them at the mean individual level. Pruning adds parsimony to the model even after accounting for loss aversion (cf. ‘Discount & Loss’ vs ‘Pruning & Loss’), while loss aversion does not increase parsimony when added to the best previous model (‘Pruning & Learned’ vs ‘Loss & Prune & Learned’). **B**: Separate inference of all reinforcement sensitivities from best loss aversion model. **C**: Absolute ratio of inferred sensitivity to maximal punishment (−70, −100 or −140) and inferred sensitivity to maximal reward (always +140). Subjects are 1.4 times more sensitive to punishments than to rewards.

To distinguish loss aversion from pruning, we fit several additional models. Model ‘Loss’ is equal to model ‘Look-ahead’ in that it assumes that subjects evaluate the entire tree. It differs, in that it infers, for every subject, what effective weight they assigned each reinforcement. In the above example, for the overall sequence to be as subjectively bad as if the reinforcement behind it had been neglected, the −100 reinforcement could be increased to an effective value of −240. By itself, this did not provide a parsimonious account of the data, as model ‘Loss’ performed poorly (Figure 5A). We augmented model ‘Loss’ in the same manner as the original model by allowing for discounting and for specific pruning. There was evidence for pruning even when reinforcement sensitivities were allowed to vary separately, i.e. even after accounting for any loss aversion (cf. models ‘Discount & Loss’ and ‘Pruning & Loss’, Figure 5A). Furthermore, adding loss aversion to the previous best model did not improve parsimony (cf. models ‘Pruning & Learned’ vs ‘Loss & Pruning & Learned’). Finally, the Pavlovian conditioned approach also provided a more parsimonious account than loss aversion (cf ‘Pruning & Learned’ vs ‘Pruning & Loss’). Replacing the four separate parameters in the ‘Loss’ model with two slope parameters to reduce the disadvantage incurred due to the higher number of parameters does not alter these conclusions (data not shown). Finally, the screen subjects saw (Figure 2A) only showed four symbols: ++, +, − and − −. It is thus conceivable that subjects treated a ++ as twice as valuable as a +, and similarly for losses. A model that forced reinforcements to obey these relationships did not improve parsimony (data not shown). The inferred reinforcement sensitivities from model ‘Pruning & Loss’ are shown in Figure 5B. Comparing the inferred sensitivities to the largest rewards and punishments showed that subjects did overvalue punishments (treating them approximately 1.4 times as aversive as an equal-sized reward was appetitive; Figure 5C), consistent with previous studies [20]. In conclusion, there is decisive evidence for specific Pavlovian pruning of decision trees above and beyond any contribution of loss aversion.

### Pruning estimates

We next examined the parameter estimates from the most parsimonious model (‘Pruning & Learned’). If subjects were indeed more likely to terminate the tree search after large punishments, and thus forfeit any rewards lurking behind them, then the specific pruning probability should exceed the general pruning probability.

**Figure 6:**
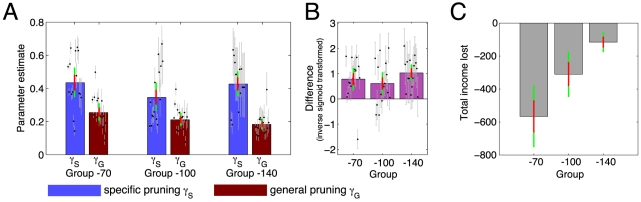
Pruning parameters. **A**: Pruning parameter estimates – specific and general pruning parameters are shown separately for each group. Specific pruning exceeded general pruning across subjects, but there was no main effect of group and no interaction. The difference between parameter types was significant in all three groups, with specific exceeding general pruning for 14/15, 12/16 and 14/15 subjects in the −70, −100 and −140 groups respectively. Blue bars show specific pruning parameters (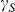) and red bars general pruning parameters (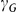). Black dots show the estimates for each subject. Gray lines show the uncertainty (square root of second moment around the parameter) for each estimate. **B**: Equivalent parametrization of the most parsimonious model to infer differences between pruning and discount factors directly. For all three groups, the difference is significantly positive. **C**: Income lost due to pruning. On trials on which the optimal sequence led through large punishments, subjects lost more income the more counterproductive pruning was (loss in group −70
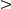
loss in group −100
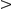
loss in group −140). Each bar shows the total income subjects lost because they avoided transitions through large losses. Throughout, the bars show the group means, with one standard error of the mean in red and the 95% confidence interval in green.

Figure 6A shows the specific and general pruning parameters 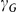 and 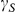 for every subject. To test for the difference we modified the parametrization of the model. Rather than inferring specific and general pruning separately, we inferred the general pruning parameter and an additional ‘specific pruning boost’, which is equivalent to inferring the difference between specific and general pruning. This difference is plotted in Figure 6B for the groups separately, though the reader is reminded that the model comparisons above did not reveal group differences (Figure 4C). The posterior probability of no difference between 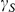 and 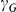 was 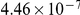.

The parsimony of separate priors was tested earlier (see Figure 4C), showing that specific pruning 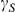 did not differ between groups. This is in spite of the fact that pruning in the groups −70 and −100 is costly, but not in the −140 group (Figure 2C). The fact that pruning continues even when disadvantageous is evidence for a simple and inflexible pruning strategy which neglects events occurring after large losses when computational demands are high. Figure 6C shows the cost of pruning in terms of the loss of income during episodes when the optimal choice sequence would have involved a transition through a large punishment. These results suggest that pruning is a Pavlovian response in the sense that it is not goal-directed and not adaptive to the task demands, but is rather an inflexible strategy reflexively applied upon encountering punishments.

### Psychometric correlates

We next tested two a priori predictions that relate the model parameters to psychometric measurements. Based on prior modelling work [17], we hypothesized that the tendency to employ the simple pruning strategy should correlate with psychometric measures related to depression and anxiety, i.e. with the BDI score and NEO neuroticism. We also expected to replicate prior findings whereby the reward sensitivity parameter 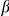 should be negatively correlated with BDI and NEO neuroticism [21]–[24]. Because parameters for different subjects were estimated with varying degrees of accuracy (see individual gray error bars in Figure 6), our primary analysis was a multiple regression model in which the influence of each subject's data was weighted according to how accurately their parameters were estimated (see Methods).

**Figure 7:**
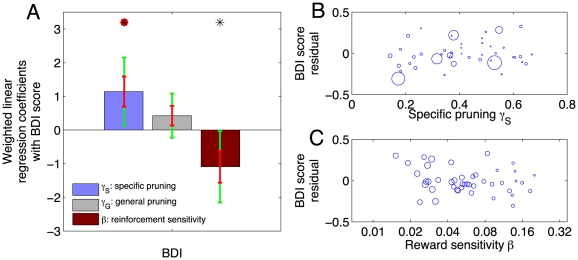
Psychometric correlates. **A**: Subclinical depression scores (Beck Depression Inventory, BDI, range 0–15) correlated positively with specific pruning (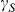), and negatively with sensitivity to the reinforcers (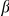). Each bar shows a weighted linear regression coefficient. Red error bars show one standard error of the mean estimate, and green errorbars the Bonferroni corrected 95% confidence interval. 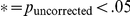, red dot 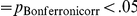. **B,C**: Weighted scatter plots of psychometric scores against parameters after orthogonalization.

We found that BDI was positively correlated with the specific pruning parameter 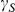 (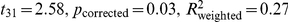). Furthermore, this effect was specific in that there was no such correlation with general pruning 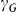. There was also a negative correlation between BDI score and reward sensitivity 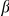, although this did not survive correction for multiple comparisons (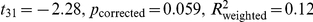). The regression coefficients for the BDI score are shown in Figure 7A. Notably, these correlations arose after correcting for age, gender, verbal IQ, working memory performance and all other NEO measures of personality. Thus, as predicted, subjects with more subclinical features of depression were more likely to curtail their search specifically after large punishment. However, against our hypothesis, we did not identify any significant correlations with NEO neuroticism.

Finally, we examined correlations between all parameters and all questionnaire measures in the same framework. We found a positive correlation between NEO agreeableness and the weight of the ‘Learned Pavlovian’ influence 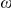 which survived full correction for 60 comparisons 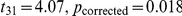.

## Discussion

We employed a Bayesian model-fitting approach to investigate how Pavlovian choices might shape goal-directed decision making. Our full model was able to account for a high percentage of subjects' choices, allowing us to draw strong conclusions about the likely forces governing their behavior. Influences were deemed Pavlovian when they were evoked in a fixed and inflexible manner in response to an outcome or a stimulus value, and goal-directed when sensitive to the ultimate, possibly distant, result of the choice [25].

Participants exhibited two highly significant Pavlovian influences. First, subjects pruned to a very substantial degree. While part of this pruning was valence independent and hence not Pavlovian (parameter 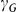 in the model), and can be seen as a natural, if suboptimal, response to the exponentially exploding complexity of complete search in the model (ranging from 2 to 256 sequences), subjects also showed a substantial increase in their propensity to prune in the face of a large negative outcome (parameter 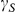 in the model). Importantly, they did so even at the expense of a substantial net loss in reward. It was striking that subjects were no less likely to prune (Figure 2C–D) even when we rendered it increasingly disadvantageous (moving from group −140 to group −70),.

The second, ‘Learned’, Pavlovian influence was associated with the learned attractiveness of previously rewarded states. In our task, states could have been associated with large rewards on past trials, but lack the potential to lead to reward (or indeed punishment) on a given trial, because insufficient choices remained (Figure 4E). Subjects were significantly seduced by the effect of these past rewards (or repulsed by punishments), again in a way that was counterproductive to optimal control. Note that by including this second Pavlovian influence, we could be sure that the pruning described above was a pure influence on goal-based evaluation, and was not corrupted by an intrinsic repulsion to the punishment (which would have been ascribed to this second, Pavlovian, influence).

The ‘Loss’ models do suggest that subjects were more sensitive to punishments than rewards (Figure 5C). However, this did not explain away pruning. Also, if the pruning we observed was just a signature of loss aversion, one would have expected the extent of pruning not to be the same across groups. Loss aversion is a specific phenomenon in behavioural economics, whereby subjects are more strongly opposed to a given probability of losing a certain amount than to winning that same amount [26]. To the extent to which loss aversion can be described as an inflexible, reactive, response to an aversive stimulus, it may represent a third instance of Pavlovian responses to losses interfering with goal-directed decisions in this task [27].

Next, subjects could transition through losses early on in the tree, but were more reluctant to do so when they appeared deeper in the tree. Pavlovian pruning thus appeared to have a particularly strong effect deeper in the tree. Although this makes intuitive sense, it is not a feature explicitly built into the models. Figure 4G shows that this *can* arise from the interaction of the particular sequence of reinforcements (and thus reinforcement depth) and the pruning and discount factors. Although this is not necessarily always the case, the fact that our best-performing model accounted so well for subjects' choices (Figure 4A) suggests that it was a sufficient mechanism for the particular set of reinforcement sequences encountered here.

Finally, although our sample of *healthy* volunteers, which was thoroughly screened for past pathology, reported only very mild depressive symptoms (with mean BDI scores of 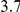, range 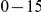), we found that subjects' propensity to prune specifically in the face of negative valence was positively correlated with self-reported sub-clinical depressive symptoms.

### Pruning, serotonin and depression

Our work was inspired by a previous modelling paper [17], which used the concept of behavioural inhibition to unify two divergent and contradictory findings on the relationship between serotonin and depression. On the one hand, drugs that putatively increase serotonin by inhibiting the serotonin reuptake mechanism are effective for both acutely treating [28], and preventing relapse of [29], depression. On the other hand, a genetic polymorphism that downregulates the very same serotonin reuptake transporter, thus acting in the same direction as the drugs, has the opposite effect on mood, predisposing towards depression and other related mood disorders ([30]; though see also [31] for a discussion of replication failures).

Dayan and Huys [17] explained this paradox by suggesting that people who experienced high levels of serotonin and thus exaggerated Pavlovian behavioural inhibition during early development [32] would be most sensitive to the effects of any interference with this inhibition in adulthood secondary to a drop in serotonin levels [33], [34]. Thus, the inhibitory consequences of serotonin could account for both its predisposing qualities on a developmental time-scale, and more acute relief during depressive episodes.

The hypothesis in [17] relates to two facets of the current study. First, if serotonin indeed mediates behavioural inhibition in the face of punishments [10], [12]–[14] then it is a strong prediction that the pruning parameter 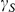, which mediates the inhibition of iterative thought processes, should be related to, and modulated by, serotonergic activity. We plan to test this directly in future studies. There is already some, though far from conclusive, evidence pointing towards such an influence of serotonin on higher-level cognition. First, serotonergic neurons project strongly to areas involved in goal-directed, affective choices including the medial prefrontal cortex [35]. Genetic variation in the serotonin transporter allele modulates functional coupling between amygdala and rostral cingulate cortex [36]. Next, orbitofrontal serotonin depletion impacts cognitive flexibility, or the adaptive ability to switch between contingencies, by impairing inhibitory control [37] in monkeys. Third, learned helplessness, which can be interpreted in goal-directed terms [17], depends critically on pre- and infralimbic cortex in rats [38], and is known to be mediated by serotonin [39]. Contrary to this, there is a recent report that mood manipulation, but not acute tryptophan depletion, impairs processing on the one-touch Tower of London (OTT) task [40], which should certainly engage goal-directed processing. One possible explanation for this apparent discrepancy is that although the OTT requires sequences of moves to be evaluated, there is no obvious aversive point at which Pavlovian pruning might be invoked. Further, although OTT is explicitly framed as a ‘cold’ task, i.e. one which does not involve affective choices, there is also supporting evidence (see below).

The second facet of our theoretical model [17] concerns depression. The model suggested that subjects prone to depression exhibit decision making that is more reliant on serotonergic function, expressed as excess pruning, but that the depressed state itself is characterised by a low serotonin state and thus a loss of pruning. The stronger dependence on serotonin in at-risk subjects would explain why only they are sensitive to the mood effects of tryptophan depletion [34], and why individuals with a polymorphism in the serotonin transporter gene that reduces serotonin uptake are more liable to develop mood disturbance, especially following serotonin depletion [41], [42]. That is, this theory predicts excessive pruning to occur in subjects *at risk* for depression, and reduced pruning to occur *during* a depressive episode. The data presented here (a positive correlation between mildly raised BDI scores and the tendency to prune when encountering a large loss; Figure 7) would be consistent with this theoretical account if mildly raised BDI scores in otherwise healthy subjects (we screened for criteria for a major depressive episode; and 94% of our participants had BDI scores 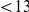, rendering depression unlikely [43]) could be interpreted as a vulnerability or proneness to depression. The mildly raised BDI scores do reveal a latent level of dysphoric symptoms amongst healthy participants [55]. This might be in line with findings that levels of dysphoric symptoms correlate with levels of dysfunctional thinking, and that a cyclical interaction between the two could, in the presence of certain environmental events, crescendo into a depressive episode proper [45], [46]. However, we are not aware of any direct evidence that mildly raised BDI scores measure vulnerability, and maybe more critically, we did not observe correlations with NEO neuroticism, which is an established risk factor for depression [47]. The strong prediction that serotonergic function and behavioural inhibition in the face of losses should be reduced during a major depressive episode remains to be tested. However, there is already some evidence in favour of this conclusion. People actively suffering from depression are impaired on the OTT [48], [49]. The impairment relative to controls grows with the difficulty of the problem; and depressed subjects also spend increasing amounts of time thinking about the harder problems, without showing improved choices [50]. This suggests that people who are suffering from depression have more difficulty searching a deep tree effectively (possibly also captured by more general, superficial autobiographical recollections; [51]). However, given the finding by [40], we note that it is at present not possible to interpret this conclusively in terms of pruning. Finally, the same group has also reported catastrophic breakdown in OTT performance in depressed subjects after negative feedback [52].

### Conclusion

We used a novel sequential decision-making task in conjunction with a sophisticated computational analysis that fitted a high proportion of healthy subjects' choices. This allowed us to unpack a central facet of effective computation, pruning. Importantly, most subjects were unable to resist pruning even when it was disadvantageous, supporting our hypothesis that this process occurs by simple, Pavlovian, behavioural inhibition of ongoing thoughts in the face of punishments [17]. Provocatively, consistent with this model, we found a relationship between the propensity to prune and sub-clinical mood disturbance, and this suggests it would be opportune to examine in detail the model's predictions that pruning should be impaired in clinically depressed individuals and following serotonin depletion.

## Methods

### Participants

Fourty-six volunteers (23 female, mean age 23.8
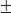
4 years) were recruited from the University College London (UCL) Psychology subject pool. Each gave written informed consent and received monetary, partially performance-dependent compensation for participating in a 1.5-hour session. The study was conducted in accord with the Helsinki declaration and approved by the UCL Graduate School Ethics Committee. Exclusion criteria were: known psychiatric or neurological disorder; medical disorder likely to lead to cognitive impairment; intelligence quotient (IQ) 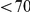; recent illicit substance use and not having English as first language. The absence of axis-I psychopathology and alcohol- or substance abuse/dependence was confirmed with the Mini International Neuropsychiatric Inventory [53]. Personality, mood, and cognitive measures were assessed with the State-Trait Anxiety Inventory [54], the Beck Depression Inventory (BDI; [55]), the NEO Personality Inventory [56], the Wechsler Test of Adult Reading (WTAR; [57]), and Digit Span [58].

Subjects who were assigned to the different groups, were matched for age, IQ and sex (all 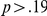, one-way ANOVA). Fifteen subjects were assigned to group −70, 16 to group −100 and 15 to group −140. Mean age (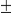
1 st. dev.) was 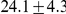, 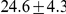 and 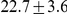 years respectively; mean digit span scores were 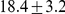, 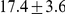 and 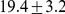; mean IQ scores (computed from WTAR) were 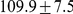, 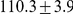 and 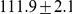. There were 5 (33%), 8 (50%) and 10 (66%) men in each of the three groups. One subjects' age information, and one subject's STAI information were lost. These subjects were excluded from the psychometric correlation analyses.

### Task

Participants first underwent extensive training to learn the transition matrix (Figure 2A,B; [16]). During the training, subjects were repeatedly placed in a random starting state and told to reach a random target state in a specified number of moves (up to 4). After 40 practice trials, training continued until the participant reached the target in 9 out of 10 trials. Most subjects passed the training criterion in three attempts. Reaching training criterion was mandatory to move on to the main task.

After training, each transition was associated with a deterministic reward (Figure 2B). Subjects completed two blocks of of 24 choice episodes; each episode included 2 to 8 trials. The first block of 24 episodes was discarded as part of training the reward matrix, and the second block of 24 episodes was analysed. At the beginning of each episode, subjects were placed randomly in one of the states (highlighted in white) and told how many moves they would have to make (i.e., 2 to 8). Their goal was to devise a sequence of that particular length of moves to maximize their total reward over the entire sequence of moves. To help the subjects remember the reward or punishment possible from each state, the appropriate “+” or “-” were always displayed beneath each box. Regardless of the state the subject finished in on a given episode, they would be placed in a random new state at the beginning of the next episode. Thus, each episode was an independent test of the subject's ability to sequentially think through the transition matrix and infer the best action sequence. After each transition, the new state was highlighted in white and the outcome displayed. On half of the trials, subjects were asked to plan ahead their last 2–4 moves together and enter them in one step without any intermittent feedback.

The reward matrix was designed to assess subjects' pruning strategy; and whether this strategy changed in an adaptive, goal-directed way. All subjects experienced the same transition matrix, but the red transitions in Figure 2C led to different losses in the three groups, of −70, −100 or −140 pence respectively. This had the effect of making pruning counterproductive in groups −70 and −100, but not −140 (Figures 2C–E). At the end of the task, subjects were awarded a monetary amount based on their performance, with a maximum of £20. They were also compensated £10 for time and travel expenses.

### Model-based analysis

In the look-ahead model, the 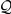-value of each action 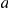 in the present state 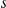 is derived by i) searching through all possible future choices; ii) always choosing the optimal option available in the future after a particular choice; and iii) assigning the two actions at the present state the values of the immediate reward plus the best possible future earnings over the entire episode. More concisely, the look-ahead (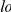) model is a standard tree search model, in which the value of a particular action is given by the sum of the immediate reward 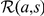 and the value of the optimal action from the next state 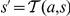


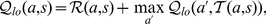
(1)where 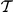 is the deterministic transition function. This equation is iterated until the end of the tree has been reached [59]. For notational clarity, we omit dependence of 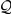 values on the depth of the tree. To make the gradients tractable, we implement the 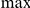 operator with a steep softmax.

An explicit search all the way to the end of the tree is unlikely for any depths 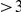, given the large computational demands. The model ‘Discount’ (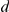) thus allowed, at each depth, a biased coin to be flipped to determine whether the tree search should proceed further, or whether it should terminate at that depth, and assume zero further earnings. Let the probability of stopping be 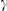. The expected outcome from a choice in a particular state, the 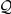 values, is now an average over all possible prunings of the tree, weighted by how likely that particular number of prunings is to occur:
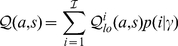
(2)where 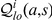 is the full lookahead value of action 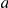 in state 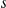 for the cut tree 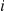. Importantly, the number 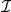 is immense. If the number of branches of a binary tree is 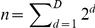, then there are 
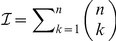
 possible ways of choosing up to 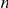 branches of the tree to cut. Although this overestimates the problem because branches off branches that have already been cut off should no longer be considered, the problem remains overly large. We therefore use a mean-field approximation, resulting in 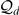 values:
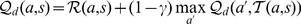
(3)where, at each step, the future is weighted by the probability 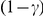 that it be encountered. This means that outcomes 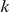 steps ahead are discounted by a factor 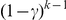. We note, however, that Equation 3 solves a different Markov decision problem exactly.

Next, the ‘Pruning’ (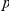) model encompassed the possibility that subjects were more likely to stop after a large punishment had been encountered. It did this by separating the stopping probability into two independent factors, resulting in:
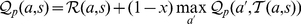
(4)

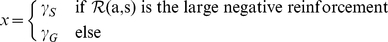
(5)where 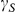 is the specific pruning parameter that denotes the probability with which the subject stops evaluation of the tree at any state-action pair associated with the large negative reward. Here, we used binary pruning rather than the graded form of [17], since there is only one extreme negative outcome. The second parameter 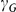 was the probability of curtailing the tree search at any other transition (−20, +20, +140) and is exactly analogous to the 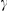 of the Discount model.

To account for ‘Learned Pavlovian’ (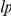) attraction or repulsion, i.e. the approach to, or avoidance of, states that are typically associated with future rewards on those trials on which these future rewards are not available (e.g. a terminal transition from state 6 to state 1), we modified the ‘Pruning’ model by adding a second state-action value which depends on the long-term experienced average value 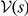 of the states:
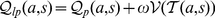
(6)The value 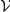 is learned by standard temporal difference learning:
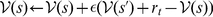
(7)where 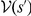 is set to zero if it is the terminal transition. This model, which we term ‘Learned + Pavlovian’, is based on [8] and the parameter 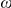 is fit to the data.

So far, when search terminates, a zero value for the rest of the decision tree was entered. An alternative to the Learned Pavlovian model is to additionally include the value 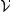 as terminal value, i.e.:
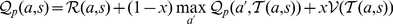
(8)with 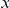 as in the Pruning model, and with 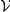 evolving as in equation 7. Note that we this model also incorporated the direct learned Pavlovian effect (Equation 6).

To account for loss aversion, we fitted models in which we inferred all reinforcement sensitivities 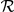 separately. Thus, these models relaxed the assumption of the above models that subjects treated a reward of 140 as exactly cancelling out a loss of −140. In fact, these models in principle allowed subjects to be attracted to a loss and repelled from a reward. We used such a free formulation to attempt to soak up as much variance as possible. If pruning is visible above and beyond this, then differential sensitivities to rewards and punishments by themselves cannot account for the pruning effects in the above models. This formulation does have the drawback that the large number of free parameters may potentially exert a prohibitive effect on the 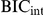 scores. Although we saw no indication of that, we fitted a further, restricted loss aversion model with two slopes, i.e. where the rewards took on values 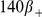 and 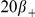, and the losses 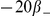 and 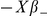. The restricted models led to the same conclusions as the full loss aversion models and we thus do not report those results.

Finally, in the habitual SARSA model, choice propensities were calculated in a model-free manner to capture habitual choices [18], [19]:
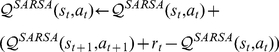
(9)


Given the 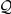 values, the probability of subjects' choices was computed as
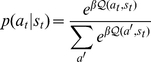
(10)where we emphasize that the 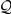 value of each choice depends on how many choices are left after 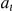, but *not* on the choices preceding it. The parameter 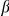 was set to unity for all loss models. We note that this probability is predictive in that it depends only on past rewards and choices, but not in the machine learning sense, whereby it predicts data not used to fit the parameters.

### Model fitting procedure

We have previously described our Bayesian model fitting and comparison approach [60], but repeat the description here for completeness. For each subject, each model specifies a vector of parameters 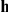. We find the maximum a posteriori estimate of each parameter for each subject: 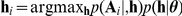 where 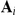 are all actions by the 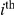 subject. We assume that actions are independent (given the stimuli, which we omit for notational clarity), and thus factorize over trials. The prior distribution on the parameters mainly serves to regularise the inference and prevent parameters that are not well-constrained from taking on extreme values. We set the parameters of the prior distribution 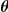 to the maximum likelihood given all the data by *all* the 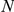 subjects:
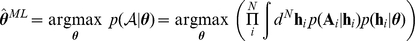
where 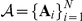. This maximisation is achieved by Expectation-Maximisation [61]. We use a Laplacian approximation for the E-step at the 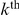 iteration:
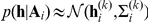


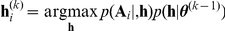
where 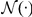 denotes a normal distribution and 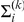 is the second moment around 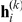, which approximates the variance, and thus the inverse of the certainty with which the parameter can be estimated. Finally, the hyperparameters 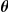 are estimated by setting the mean 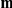 and the (factorized) variance 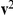 of the normal prior distribution to:
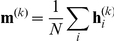


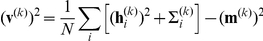
All parameters are transformed before inference to enforce constraints (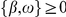, 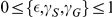).

### Model comparison

As we have no prior on the models themselves (testing only models we believe are equally likely a priori), we instead examine the model log likelihood 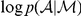 directly. This quantity can be approximated in two steps. First, at the group level [62]:
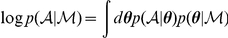


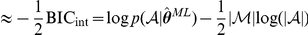
where 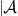 is the total number of choices made by all subjects, and 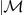 is the number of prior parameters fitted (mean and variance for each parameter). Importantly, however, 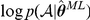 is not the sum of individual likelihoods, but the sum of *integrals* over the individual parameters (hence the subscript “int” to the Baysian Information Criterion (BIC)):
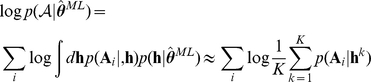
The second approximation involves replacing the integral by a sum over samples from the empirical prior 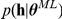. This ensures that we compare not just how well a particular model fits the data when its parameters are optimized, but how well the model fits the data when we only use information about where the group parameters lie on average.

### Statistical analysis

#### Group comparisons

We used a Bayesian model comparison approach to compare the three groups receiving different maximal punishments. To do so, we fitted models that allowed for separate prior parameters for each group, and penalized this overall model according to its 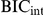 score. Here, the number 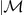 was increased to the total number group-level parameters for all groups jointly.

#### Correlation analyses

We used a weighted hierarchical multivariate regression, which is equivalent to a standard hierarchical multivariate regression, except that parameters were weighted by the precision with which they were estimated. As this is, to our knowledge, non-standard, we describe it in some detail.

The first step consisted of a sequential orthogonalization procedure of the questionnaire measures, whereby we entered the measurements in the following sequence: 1. Age, 2. Sex, 3. IQ (computed from WTAR), 4. Digit Span, 5. NEO E, 6. NEO O, 7. NEO A, 8. NEO C, 9. STAI Trait, 10. STAI State, 11. NEO N, 12. BDI, with the consequence that regressors entered later only retained variation along dimensions orthogonal to the previously entered regressors. We then seeked regression coefficients such that
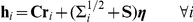
where 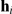 is the parameter vector for subject 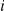, 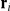 is the vector of orthogonalized psychometric measures for that subject, and 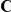 is the regression matrix we seek to infer. Crucially, two sources of noise are assumed to contribue. First, 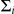 is the uncertainty about the inferred value 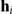. This is noise that originates from the model-based estimation procedure (i.e. at the within-subject level). Second, 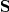 is a diagonal matrix the components 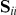 of which are the standard regression noise (capturing noise at the between-subject level) for each of the five model parameters. Including both terms, rather than just the latter, means that parameters that are better constrained by the behavioural data contribute more to the inference. This reduces to multiple multivariate linear regression if the 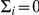. To perform the inference, the above set of 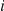 vector equations are written in terms of normal log likelihoods:
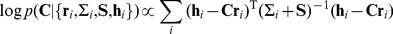
(11)This can be rewritten such as to yield one quadratic likelihood in the concatenation 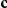 of all columns of 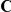, and can then be solved for both 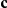 and 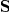 by gradient ascent.

To ascertain significance, we computed a t-statistic for each coefficient 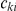. To do so, we replaced the estimate of the sum squared error in a standard t-statistic [63] with our estimate of the between-subject variance 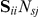 for each of the 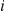 model parameters, yielding:
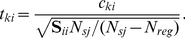
(12)Corresponding 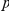-values were then calculated from the inverse cumulative Student's 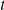-distribution as in a standard multiple regression model assuming 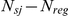 degrees of freedom, where 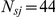 is the number of subjects for which all measurements were present, 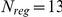 is the number of psychometric regressor variables (12 plus one constant regressor). These 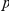-values were then thresholded at a Bonferroni-corrected level 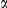 corresponding to two independent comparisons (for correlation with BDI score and with NEO neuroticism) score.
